# Refractive, Topographic, and Aberrometric Results at 2-Year Follow-Up for Accelerated Corneal Cross-Link for Progressive Keratoconus

**DOI:** 10.1155/2017/5714372

**Published:** 2017-01-18

**Authors:** Ercüment Bozkurt, Engin Bilge Ozgurhan, Betul Ilkay Sezgin Akcay, Tugba Kurt, Yusuf Yildirim, Zehra Karaagaç Günaydin, Ahmet Demirok

**Affiliations:** ^1^Beyoglu Eye Training and Research Hospital, Department of Ophthalmology, Istanbul, Turkey; ^2^Umraniye Training and Research Hospital, Department of Ophthalmology, Istanbul, Turkey; ^3^Istanbul Medeniyet University Medical School, Department of Ophthalmology, Istanbul, Turkey

## Abstract

*Purpose*. To report the visual, refractive, and corneal topography and wavefront aberration results of accelerated corneal cross-linking (CXL) during a 24-month follow-up.* Methods*. Forty-seven eyes underwent riboflavin-ultraviolet A-induced accelerated CXL treatment (30 mW/cm^2^ with a total dose of 7.2 joules/cm^2^). Uncorrected distance visual acuity (UDVA), corrected distance visual acuity (CDVA), spherical and cylindrical values, keratometry (*K*) measurements (*K*_steep_, *K*_flat_, *K*_avg_, and *K*_apex_), central corneal thickness, and anterior corneal aberrometric analyses including total wavefront error (WFE), total high order aberration (HOA), astigmatism, trefoil, coma, quadrafoil, secondary astigmatism, and spherical aberration were evaluated.* Results*. The mean UDVA and CDVA were significantly improved at 1 (*p* = 0.003 and *p* = 0.004, resp.) and 2 years after treatment (*p* = 0.001 and *p* = 0.001, resp.). The mean *K*_steep_, *K*_flat_, *K*_average_, and *K*_apex_ values were significantly lower than baseline at 12 months (*p* = 0.008, *p* = 0.024, *p* = 0.001, and *p* = 0.014, resp.) and 24 months (*p* = 0.014, *p* = 0.017, *p* = 0.001, and *p* = 0.012, resp.). Corneal thickness showed a significant decrease at 1 month. Total HOA and coma decreased significantly at the 12-month (*p* = 0.001 and *p* = 0.009, resp.) and 24-month visits (*p* = 0.001 and *p* = 0.007, resp.).* Conclusion*. Accelerated CXL (30 mW/cm^2^) was found to be effective in improving UDVA, CDVA, corneal topography readings, total HOA, and coma aberrations during the 24-month follow-up.

## 1. Introduction

Riboflavin and ultraviolet A (UVA) induced corneal cross-linking (CXL) is an effective treatment for progressive keratoconus and has gained popularity since it has been introduced by Wollensak et al. in 2003 [1]. The standard CXL treatment uses UVA light with 3 mW/cm^2^ radiation for a duration of 30 min, corresponding to a total energy dose of 5.4 J/cm^2^ [1]. More recently, accelerated corneal CXL that uses higher energy settings (up to an irradiance of 30 mW/cm^2^) has gained popularity owing to its shorter treatment duration (3–10 min). Bunsen-Roscoe law (the reciprocity law) is the fundamental law of photochemistry that states that similar biological effects can be produced with long illumination time and low irradiation intensity by reducing illumination time and increasing radiation intensity (i.e., 10 min of illumination at 9.0 mW/cm^2^ provides the same effect as 30 min of illumination at 3.0 mW/cm^2^, with a total surface dose of 5.4 J/cm^2^ in both cases) [2]. Preclinical studies in porcine corneas have revealed that accelerated CXL induces equivalent biomechanical changes as those induced by standard treatment [3, 4]. There are limited reports on the effects of administering accelerated CXL with a total dose of 7.2 J/cm^2^ [5]. Here, we report the visual, refractive, corneal topography, and wavefront aberration results of this treatment in a 24-month follow-up examination.

## 2. Materials and Methods

This study was performed in accordance with the Declaration of Helsinki and approved by the local ethics committee of the Beyoglu Eye Research and Education Hospital. The patients were informed about the potential risks and benefits of all procedures and written informed consent was obtained in accordance with institutional and legal requirements before any surgical intervention.

This study design was a retrospective chart review, which included 47 eyes of 41 patients with keratoconus undergoing accelerated CXL. The included eyes showed mild-to-moderate keratoconus (grades I-II by the Amsler-Krumeich classification). Progression of ectasia was defined as an increase in manifest refraction spherical equivalent ≥ 0.50 D, *K*_max_ increase ≥ 1 diopter (D), and corneal astigmatism increase ≥ 1 D, confirmed by consecutive examinations at 3-month intervals. Exclusion criteria were as follows: preoperative pachymetry < 400 mm, maximum keratometry value (*K*_max_) > 65 D, history of ocular surgery, corneal scars, history of herpetic keratitis, coexisting ocular pathology, connective tissue disease, pregnancy or nursing status, and systemic medication use that would likely affect corneal wound healing. Patients were instructed to discontinue the use of hard contact lenses at least 2 weeks prior to accelerated CXL treatment. Comprehensive examinations were performed in all cases, including uncorrected distance visual acuity (UDVA) and corrected distance visual acuity (CDVA), manifest refraction, slit-lamp biomicroscopy, Goldmann tonometry, and dilated fundus examination. Corneal topographic and corneal anterior wavefront aberration analyses were performed using a Scheimpflug photography-based topography system (Sirius system, Costruzioni Strumenti Oftalmici, Italy). Corneal wavefront errors were analysed over a 3 mm optical zone and decomposed into Zernike polynomials up to the sixth order. The parameters analysed included total wavefront error (WFE), total high order aberration (HOA), astigmatism, trefoil, coma, quadrafoil, secondary astigmatism, and spherical aberration. Visual acuity measurements included uncorrected distance visual acuity (UDVA) and corrected distance visual acuity (CDVA) and were assessed with the early treatment diabetic retinopathy study logarithm of the minimal angle of resolution (LogMAR) charts.

The primary outcome measures of the study were UDVA, CDVA, spherical and cylindrical values, keratometry (*K*) measurements (*K*_steep_, *K*_flat_, *K*_average_, and *K*_apex_), central corneal thickness, and corneal anterior wavefront aberration analyses. These were evaluated at baseline and at each of the postoperative follow-up examinations (1, 6, 12, and 24 months).

### 2.1. Surgical Technique

CXL was performed under sterile conditions in the operating room. After applying topical anesthesia with proxymetacaine hydrochloride (0.5%) eye-drops (Alcaine, Alcon Co. Inc., Canada), the corneal epithelium was removed with a blunt spatula (8.0–9.0 mm diameter). Riboflavin (0.1% solution VibeX; Avedro Inc., Waltham, MA) was instilled at the center of the cornea for 15 min (one drop every 2 min). The cornea was exposed to 365 nm UVA light with the CXL system (Avedro Inc., Boston, USA) for 4 min at an irradiance level of 30 mW/cm^2^ (total surface dose, 7.2 J/cm^2^). A soft contact lens with a 14.0 mm diameter, 8.6 base curvature, and oxygen permeability of 140 barrers (lotrafilcon B [Air Optix], Alcon Laboratories Inc.) was applied at the end of the procedure. Postoperatively, moxifloxacin (0.5%, Vigamox, Alcon, Inc. Canada) was applied 4 times per day for 1 week, and artificial tears were administered 4 times per day for 1 month, at which point the soft contact lenses were removed. Patients received fluorometholone acetate (0.1%, Flarex, Alcon Inc., Canada) 4 times per day, which was tapered down over 2 weeks after removal of the soft contact lens. All patients were followed up daily until the epithelium healed.

### 2.2. Statistical Analysis

Statistical analysis was performed using the Statistical Package for the Social Sciences software version 11.5 (SPSS Inc., Chicago, IL, USA). Data were expressed as mean ± standard deviation (SD). The Kolmogorov-Smirnov test was used to assess the normality of all data samples. A paired* t*-test was used to compare the postoperative changes with the preoperative values. Differences with a value of *p* < 0.05 were considered statistically significant.

## 3. Results

Forty-seven eyes of 41 patients (20 females, 21 males; mean age, 23.87 ± 5.07 years; age range was 18–34 years) underwent treatment in this study. Most of the patients reported mild-to-moderate pain, which was relieved 2–4 days following the procedure. The duration of reepithelization ranged between 2 and 4 days. No postoperative complications (e.g., infection or corneal haze) were observed.

### 3.1. Visual, Refractive, and Topographic Outcomes

**Figure 1:**
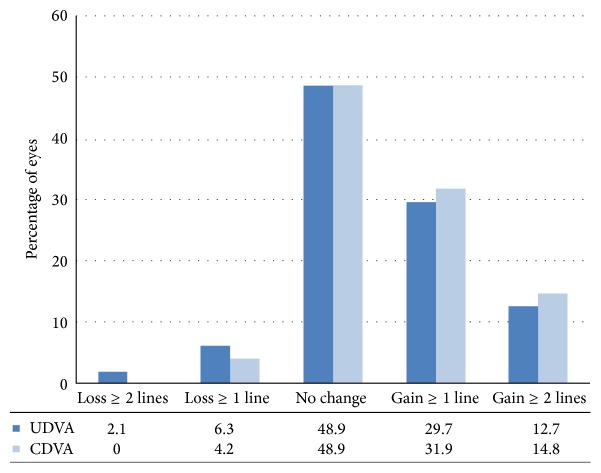
Uncorrected (UDVA) and corrected (CDVA) distance visual acuity changes before and 24 months after accelerated corneal cross-linking.

**Figure 2:**
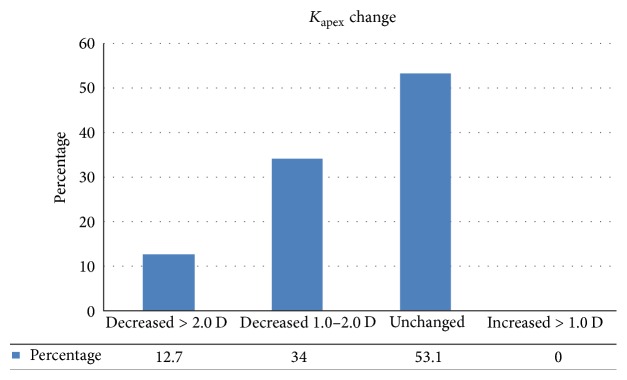
Changes in maximum simulated keratometry (*K*_apex_) values before and 1, 6, 12, and 24 months after accelerated corneal cross-linking.

Postoperative visual and refractive results are shown in Table 1. There was no significant change in mean UDVA and CDVA at 1 month (*p* = 0.345 and *p* = 0.148, resp.) and 6 months (*p* = 0.653 and *p* = 0.274 resp.) postoperatively. The mean UDVA and CDVA were significantly improved at 12 months (*p* = 0.003 and *p* = 0.004, resp.) and 24 months (*p* = 0.001 and *p* = 0.001, resp.) as compared to baseline values. At 24 months, 12.7% of the eyes gained at least 2 lines of the UDVA, 29.7% of eyes gained 1 line, 48.9% displayed no change, 6.3% lost 1 line, and 2.1% lost 2 lines. While 14.8% of the eyes gained at least 2 lines of the CDVA, 31.9% gained 1 line, 48.9% had no change, and 4.2% lost 1 line (Figure 1). The mean spherical and cylindrical refraction were not significantly changed at each visit after CXL as compared to baseline values (*p* > 0.05 for all visits). The mean *K*_steep_, *K*_flat_, *K*_apex_, and *K*_average_ values were not significantly changed at the 1- and 6-month visits (*p* > 0.05 for all) but were significantly lower than baseline at the 12-month (*p* = 0.008, *p* = 0.024, *p* = 0.001, and *p* = 0.014, resp.) and 24-month visits (*p* = 0.014, *p* = 0.017, *p* = 0.001, and *p* = 0.012, resp.). At the 24-month follow-up, *K*_apex_ values remained stable in 53.1% of eyes, decreased by 1-2 D in 34% of eyes, and decreased more than 2 D in 12.7% eyes (Figure 2). No eye showed an increase of *K*_apex_ greater than 1 D at 24 months. Corneal thickness showed a significant decrease at the 1-month visit (*p* = 0.002) but no significant change at the 6-, 12-, and 24-month visits, compared to baseline values (*p* = 0.203, *p* = 0.104, and *p* = 0.71, resp.).

### 3.2. Corneal Wavefront Aberration

Table 2 shows the corneal wavefront aberration changes in the study population. Total HOA decreased significantly at 12 and 24 months relative to baseline (*p* = 0.001 for both). Coma also decreased significantly at 12 and 24 months compared to baseline values (*p* = 0.004 and *p* = 0.007), respectively. Total wavefront error, astigmatism, trefoil, quadrafoil, secondary astigmatism, and spherical aberration values were not significantly changed at any of the postoperative visits when compared to baseline values (*p* > 0.05 for all).

## 4. Discussion

Advanced medical instrumentation with higher energy power settings has allowed CXL treatment time to be shortened. Higher energy settings (up to an irradiance of 30 mW/cm^2^) in conjunction with shorter treatment duration (3–10 min) are used in accelerated corneal CXL to maintain the same total radiant exposure as used in standard CXL. Previous studies with settings of 30 mW/cm^2^ for 3 min or 9 mW/cm^2^ for 10 min (each with a total dose of 5.4 joules/cm^2^) have shown encouraging results for accelerated CXL treatment of progressive keratoconus [6–11]. This study assessed the 24-month outcomes of accelerated CXL with a total dose of 7.2 J/cm^2^ for treatment of progressive keratoconus.

In our study, 14.8% of the eyes gained at least 2 lines of the CDVA, 31.9% gained 1 line, 48.9% had no change, and 4.2% lost 1 line at the end of 24 months of follow-up time. The mean UDVA and CDVA were improved significantly at 12 and 24 months after treatment as compared with baseline measurements (*p* < 0.05). Ozgurhan et al. have evaluated the one-year results of accelerated CXL (total dose, 7.2 J/cm^2^) on thin keratoconic corneas [11]. They found a nonsignificant improvement in UDVA and CDVA during 12 months of follow-up. Waszczykowska and Jurowski have evaluated the results of accelerated CXL at 6 mW/cm^2^ during two years' follow-up [12]. Contrary to our findings, their results did not show significant improvements in UDVA and CDVA from baseline to the 12th and 24th months. Elbaz et al. have evaluated the one-year result of accelerated cross-linking (irradiance of 9 mW/cm^2^; 10 min) in keratoconus-affected eyes and did not find statistically significant changes in the mean CDVA; on the other hand, they found significant improvement in the mean UDVA (*p* = 0.012) at 12 months [10]. Mita et al. have evaluated the effectiveness of accelerated corneal CXL with riboflavin in keratoconus over 6-month follow-up and found a statistically significant improvement in UDVA (*p* < 0.05) [9].

In the current study, the mean *K*_steep_, *K*_flat_, *K*_apex_, and *K*_average_ values were significantly lower than baseline at the 12- and 24-month visits (*p* < 0.05 for all), which are consistent with previous results from Ozgurhan et al. [11]. Waszczykowska and Jurowski and Elbaz et al. did not observe statistically significant differences in *K*_steep_, *K*_flat_, *K*_average_, or corneal astigmatism values at any stage of their studies [10, 12]. On the other hand, Mita et al. have demonstrated a significant decrease in *K*_max_ readings at 6 months following accelerated CXL (*p* < 0.05) [9].

Corneal high order aberrations, especially coma, are important optical quality parameters and should be considered as a tool for monitoring the efficacy of keratoconus patients' treatments. Recent studies have shown increased presence of spherical and coma-like aberrations in eyes with keratoconus compared to normal controls [13, 14]. We found that total HOA and coma were significantly decreased at the 12- and 24-month visits compared to baseline. However, total wavefront error, astigmatism, trefoil, quadrafoil, secondary astigmatism, and spherical aberration values were not significantly changed at any of the postoperative visits. Ozgurhan et al. found that the total HOA, coma, and secondary astigmatism values decreased significantly at 6, 12, and 24 months following treatment in pediatric patients with keratoconus [5]. Ghanem and Caporossi et al. reported significant reduction in corneal high order aberrations 24 months after performing CXL for progressive keratoconus [2, 15].

Corneal thinning, postoperative dehydration, and alterations in epithelial healing and distribution are all commonly seen immediately after CXL procedure [16–18]. We found that corneal thickness significantly decreased 1-month postoperatively and returned to baseline value by the 6-month follow-up. Similarly, recent studies have shown that corneal thickness gradually decreases within the first 6 months following CXL and returns to baseline values by 1 year following standard CXL [18, 19].

Mazzotta et al. compared the results of pulsed and continuous accelerated cross-linking. They hypothesized that pulsed light treatment might provide higher oxygen availability than continuous light treatment [20], though there is currently no laboratory study or long-term clinical follow-up supporting this hypothesis.

The corneal-stromal demarcation line is the region of transition from normal keratocytes into elongated, hyperreflective, and needlelike structures and then into an area of large hyperreflective stromal bands. The corneal-stromal demarcation line is considered to be a marker, which can be detected by either anterior segment optical coherence tomography or confocal microscopy, for monitoring the effective depth of CXL treatment, though there is yet no evidence supporting this hypothesis [21, 22].

Our study has some limitations that include its retrospective design, lack of a demarcation line-depth evaluation, and the absence of a control group, which would have undergone different treatment settings.

In conclusion, our results are encouraging and in accordance with both published studies on standard CXL and with recently published studies on the effects of accelerated CXL throughout a 24-month follow-up period. Longer follow-up studies using various energy settings in larger cohorts are needed to validate these findings.

## Figures and Tables

**Table 1:** Visual, refractive, and corneal topography data before and 1, 6, 12, and 24 months after accelerated corneal cross-linking.

Characteristics (mean ± SD)	Preoperative	1 M	6 M	12 M	*p* ^1^	24 M	*p* ^2^
UDVA (LogMAR)	0.56 ± 0.38	0.57 ± 0.33	0.50 ± 0.29	0.47 ± 0.27	0.003^**∗**^	0.46 ± 0.29	0.001^**∗**^
CDVA (LogMAR)	0.42 ± 0.26	0.42 ± 0.25	0.36 ± 0.25	0.34 ± 0.21	0.004^**∗**^	0.33 ± 0.22	0.001^**∗**^
Spherical values (D)	−3.79 ± 2.45	−3.78 ± 2.51	−3.72 ± 2.42	−3.53 ± 2.58	**0.143**	−3.50 ± 2.54	**0.096**
Cylindric values (D)	3.75 ± 1.76	3.81 ± 1.77	3.70 ± 1.74	3.72 ± 1.63	**0.806**	3.66 ± 1.58	**0.458**
*K*_steep_ (D)	46.14 ± 2.71	46.28 ± 2.88	46.16 ± 2.80	45.94 ± 2.87	0.008^**∗**^	45.99 ± 2.74	0.014^**∗**^
*K*_flat_ (D)	49.97 ± 3.60	50.31 ± 3.75	49.87 ± 3.56	49.63 ± 3.56	0.024^**∗**^	49.62 ± 3.47	0.017^**∗**^
*K*_average_ (D)	47.88 ± 3.00	48.10 ± 3.17	47.93 ± 3.04	47.70 ± 3.07	0.014^**∗**^	47.72 ± 2.94	0.012^**∗**^
*K*_apex_ (D)	56.40 ± 4.55	56.63 ± 4.45	56.34 ± 4.63	55.68 ± 4.67	0.001^**∗**^	55.53 ± 4.54	0.001^**∗**^
Central corneal thickness (*μ*m)	443.036 ± 41.24	428.10 ± 44.78	439.17 ± 44.10	445.21 ± 43.68	0.104	446.68 ± 30.03	0.071

SD: standard deviation, M: month, UDVA: uncorrected distance visual acuity, CDVA: corrected distance visual acuity, *K*: keratometry, D: diopter, *p* ^1^, comparison of preoperative and 12th month values, *p* ^2^, comparison of preoperative and 24th month values, and ^*∗*^statistically significant increased values (*p* < 0.05).

**Table 2:** Corneal wavefront aberrations before and 1, 6, 12, and 24 months after accelerated corneal cross-linking.

Characteristics (mean ± SD)	Preoperative	1 M	6 M	12 M	*p* ^1^	24 M	*p* ^2^
Total WFE	1.36 ± 0.59	1.38 ± 0.56	1.34 ± 0.56	1.37 ± 0.54	**0.986**	1.30 ± 0.56	**0.116**
Total HOA	0.54 ± 0.26	0.53 ± 0.25	0.51 ± 0.28	0.50 ± 0.25	0.001^**∗**^	0.50 ± 0.26	0.001^**∗**^
Astigmatism	1.23 ± 0.57	1.25 ± 0.55	1.21 ± 0.53	1.25 ± 0.56	**0.582**	1.18 ± 0.55	**0.073**
Trefoil	0.17 ± 0.10	0.17 ± 0.10	0.17 ± 0.10	0.16 ± 0.09	**0.505**	0.16 ± 0.11	**0.459**
Coma	0.45 ± 0.26	0.45 ± 0.25	0.43 ± 0.26	0.42 ± 0.25	0.009^**∗**^	0.42 ± 0.25	0.007^**∗**^
Quadrafoil	0.05 ± 0.03	0.06 ± 0.05	0.06 ± 0.03	0.06 ± 0.04	**0.406**	0.06 ± 0.04	**0.237**
Secondary astigmatism	0.08 ± 0.05	0.07 ± 0.04	0.07 ± 0.06	0.06 ± 0.05	**0.062**	0.07 ± 0.05	**0.072**
Spherical aberration	0.08 ± 0.06	0.08 ± 0.06	0.07 ± 0.06	0.07 ± 0.07	**0.084**	0.07 ± 0.06	**0.060**

SD: standard deviation, M: month, WFE: wavefront error, HOA: high order aberrations, *p* ^1^, comparison of preoperative and 12th month values, *p* ^2^, comparison of preoperative and 24th month values, and ^**∗**^statistically significant increased values (*p* < 0.05).
